# Regulation of neuropeptide Y in body microenvironments and its potential application in therapies: a review

**DOI:** 10.1186/s13578-021-00657-7

**Published:** 2021-08-03

**Authors:** Yan Zhang, Chu-Yun Liu, Wei-Can Chen, Yan-Chuan Shi, Cong-Mei Wang, Shu Lin, He-Fan He

**Affiliations:** 1 Department of Anesthesiology, The Second Affiliated Hospital of Fujian Medical University, No. 34 North Zhongshan Road, Quanzhou, 362000 Fujian China; 2 Diabetes and Metabolism Division, Garvan Institute of Medical Research, 384 Victoria Street, Darlinghurst, Sydney, NSW 2010 Australia; 3 Centre of Neurological and Metabolic Research, The Second Affiliated Hospital of Fujian Medical University, Quanzhou, Fujian China

**Keywords:** Neuropeptide Y, Microenvironments, Neurotrophic factors

## Abstract

Neuropeptide Y (NPY), one of the most abundant neuropeptides in the body, is widely expressed in the central and peripheral nervous systems and acts on the cardiovascular, digestive, endocrine, and nervous systems. NPY affects the nutritional and inflammatory microenvironments through its interaction with immune cells, brain-derived trophic factor (BDNF), and angiogenesis promotion to maintain body homeostasis. Additionally, NPY has great potential for therapeutic applications against various diseases, especially as an adjuvant therapy for stem cells. In this review, we discuss the research progress regarding NPY, as well as the current evidence for the regulation of NPY in each microenvironment, and provide prospects for further research on related diseases.

## Background

Neuropeptide Y (NPY), a 36-amino acid polypeptide belonging to a family that includes peptide YY and pancreatic polypeptides [1], is expressed throughout the central (CNS) and peripheral nervous systems (PNS) [2, 3]. Sympathetic neurons release NPY with norepinephrine to coordinate parasympathetic and sympathetic neurotransmission [4]. NPY regulates brain activity, resilience to stress, digestion, blood pressure, heart rate, body metabolism, and immune functions [5]. Studies have found that NPY is neuroprotective, restores bone marrow dysfunction, and regulates bone marrow microenvironment composition [6].

NPY binds to NPY receptors (NPYRs), which belong to the class A or rhodopsin-like G-protein coupled receptor family [1, 7, 8]. To date, five NPYRs (Y1R, Y2R, Y4R, Y5R, and Y6R) have been cloned from mammals, of which only four (hY1R, hY2R, hY4R, and hY5R) function in humans [9] (Table 1).

**Table 1:** Distribution and function of NPY receptor subtypes in humans

Subtypes	Agonists	Antagonists	Distribution	Functions	References
Y1	[Phe7, Pro34]NPY, [Pro34] NPY [Leu31, Pro34] NPY	BIBP3226, BIBO3304, GR231118, BMS-193885, SR120819	Hypothalamus, spinal cord, tonsils, vascular smooth muscle, sympathetic nerve endings	Immunoreactivity, vasoconstriction, anxiolytic antidepressant, analgesia, antiepileptic, neuroprotection	[24]
Y2	TM30338 PYY (3–36), NPY (13–36)	JNJ-5207787, BIIE0246 T 4-[NPY 33–36]	Hippocampus, intestinal mucosa, amygdala, hypothalamus, sympathetic nerve endings, lateral nucleus, brainstem	Anxiolytic, antiepileptic, neuroprotector, appetite regulator, antinociceptive, anorexia, bone formation	[25]
Y4	1229U91,PP, TM30338		Hypothalamus, skeletal muscle, coronary artery, pancreas, kidney, lung	Inhibits exocrine secretions, relaxes gall bladder, stimulates luteinizing hormone secretion, regulates food intake, protects neurons	[26]
Y5	[Ala 31, Aib 32] NPY CPP1–7, NPY19–23	MK- 0557, Velneperit, L-152,804	Central nervous system, hippocampus, hypothalamus specific areas	Excites hippocampal activity, regulates circadian rhythm, anticonvulsant, anxiolytic, neuroprotection	[25, 26]

Y1R mRNA has been detected in rat pancreatic β cells [10] and colon [11], and in visceral adipose tissues [12]. In humans, Y1R is expressed throughout the colonic epithelium, mucosal nerve, heart, adrenal gland, kidney, and placenta [13]. Y2R is expressed throughout rodent, canine, and gibbon kidneys [14]. Human Y2Rs are widely distributed in presynaptic neurons and regulate neurotransmitter release [15, 16]. The Y4R comprises 375 amino acids and has been found in the gastrointestinal tract [17, 18], hippocampus, hypothalamus, pancreas, prostate, and epidermis [18–22]. Y5R is well conserved in mammals and expressed predominantly in CNS structures, including the thalamus, temporal cortex, hypothalamus, and amygdala [23].

Studies have shown that NPY can affect the microenvironment by acting on different receptors in different cells. Microenvironments include inflammatory microenvironments and nutrient microenvironments. Under physiological circumstances, somatic cells require essential nutrients to maintain their survival, growth, and differentiation [27]. When foreign bacteria invade, somatic cells activate an inflammatory response to defend against pathogens and maintain the local microenvironment's homeostasis.

The inflammatory microenvironment comprises stromal cells, including macrophages, vascular endothelial cells, adipocytes and their secreted cytokines, and the extracellular matrix. This is similar to the hematopoietic microenvironment [28], in which, too, various types of cells have been identified, including osteoblasts, stromal cells, vascular endothelial cells, macrophages, adipocytes, and megakaryocytes [28].

The nutritional microenvironment affects various cell functions, and the secretion of factors that modulate tissue behaviour, including the production of various nutritional factors, promotion of angiogenesis, and transportation of substances essential for survival. Previous studies have revealed that NPY regulates immune cell homeostasis, bone homeostasis, and vascular remodelling by activating the NPYRs expressed in macrophages, osteoblasts, and endothelial cells [2, 29, 30]. Here, we focus on how NPY and its receptors influence the microenvironments of various cells and how they regulate the synthesis and release of various cytokines.

## NPY and the inflammatory microenvironment

Inflammation is a complex process involving joint participation by the nervous, endocrine, and immune systems; it is precisely regulated. The PNS regulates inflammation through the sympathetic and parasympathetic nerves [31–33]. Several cells are involved in the inflammatory response, such as granulocytes, macrophages, and lymphocytes. Furthermore, NPY can be secreted by sympathetic neurons to regulate inflammation. Sympathetic neurons can mobilise immune cells to a specific site and stimulate their secretion of cytokine [34], which have immuno-neuro-endocrine modulatory functions.

### NPY and inflammatory cells

#### Macrophages

Macrophages are critical constituents of the immune system. They differentiate from peripheral blood mononuclear cells and are distributed throughout almost all tissue types. Activated macrophages induce localised inflammation by secreting tumour necrosis factor (TNF)-α, interleukin (IL)-1β, IL-6, high mobility group protein B1, leukotrienes, prostaglandins, elastases, lysozymes, urokinases, and matrix metalloproteinases [35, 36]. The cytokines secreted by activated macrophages mobilise granulocytes and lymphocytes to participate in local and systemic inflammatory responses.

Interestingly, NPY is a potent regulator of inflammation. It affects cell adhesion, migration, monocyte/macrophage phagocytosis, and cytokine secretion [37–40]. Among the NPY receptor subtypes, Y1R plays a crucial role in regulating inflammation, and Y1R is highly expressed by macrophages [29]. NPY can regulate the activity of macrophages and induce them to synthesise and secrete IL-4, IL-12, TNF-α, and nitrogen oxides [41].

Choi et al. found that pentraxin 3 (PTX3) mediates NPY expression and the recruitment of macrophages to specific sites in the body [42]. Both TNF-α and IL-1β induce the release of PTX3 protein from vascular smooth muscle cells (VSMCs). These results suggest that PTX3 may induce macrophage chemotaxis to VSMCs by upregulating NPY in VSMCs and NPYR expression in macrophages [42]. The Y1R antagonist BIBO3304 blocked the PTX3-mediated promotion of macrophage chemotaxis at 200 nm, supporting the idea that PTX3 promotes macrophage chemotaxis by activating NPYR signalling [42].

Our research group showed that NPY bound to macrophage Y1Rs, which induced MMP-8 expression and regulated macrophage ERK1/2 activity. NPY increased the phosphorylation of ERK1/2, and this phosphorylation was markedly attenuated upon co-treatment with the Y1R antagonist. Alternatively, PD98059, an ERK1/2 inhibitor, reduced NPY-induced MMP-8 expression. Additionally, NPY promoted macrophage migration in vitro [43]. Park et al. demonstrated that NPY induces macrophage PI3K/Akt/mTOR/eIL4E signalling pathway activation, which results in the production of transforming growth factor (TGF), leading to the upregulation of TGF-β expression and secretion by macrophages [44]. Moreover, Y2R and Y5R activation also regulated macrophage induction of inflammation [45].

**Fig. 1:**
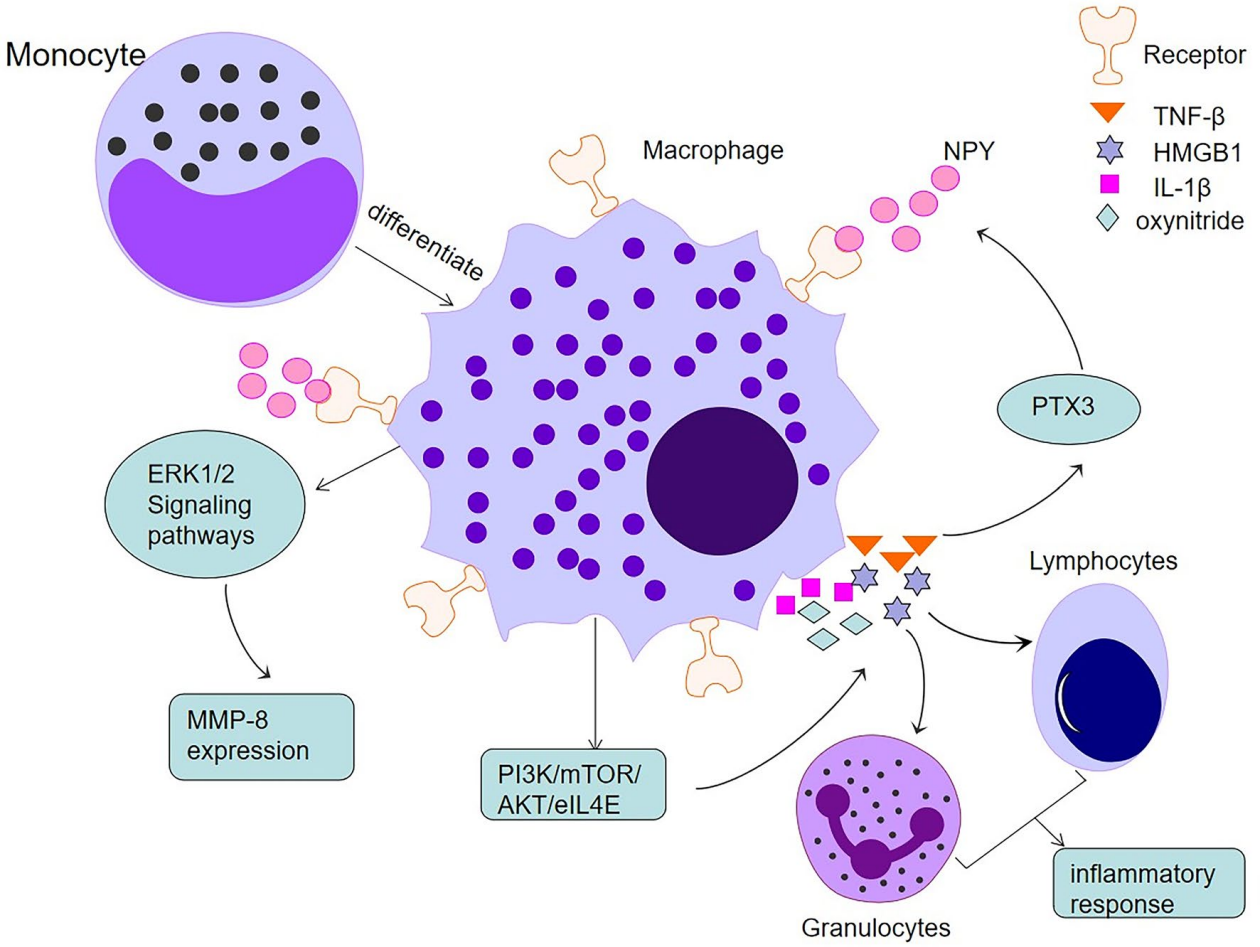
Macrophage-mediated inflammatory response. Monocyte-differentiated macrophages secrete various inflammatory factors acting on granulocytes and lymphocytes to mediate the inflammatory response. Meanwhile, inflammatory factors acting on PTX3 chemokine and ERK1/2 signalling pathways further promote macrophage migration by stimulating macrophage recruitment and MMP-8 expression via NPY receptors on macrophages, respectively

In summary, NPY affects the immune microenvironment by inducing macrophages to secrete substances that affect cell adhesion, migration, phagocytosis, and the release of cytokines by monocytes, including macrophages (Fig. 1).

#### Granulocytes

Conventionally, granulocytes include neutrophils, eosinophils, and basophils [46]; neutrophils are mainly involved in inflammation induction and play a significant role in the early inflammation stages.

Stephan et al. demonstrated that the number of granulocytes in rat blood increased significantly 15 min after intravenous injection of NPY [47]. Behavioural changes were also observed in rats with Y1R-specific inhibition of blood granulocyte function and non-Y1R-mediated stimulation of granulocyte function [47]. Katarina et al. reported that 10^−6^ M NPY enhanced the adhesiveness of peripheral granulocytes stimulated by LPS in vitro. In contrast, NPY3-36 (a Y2R and Y5R agonist) and hAAib-NPY (a Y5R agonist) significantly reduced granulocytes' adhesion ability. Also, BIBO3304, BIE0246, and L152804, specific antagonists of Y1R, Y2R, and Y5R, antagonised the respective NPY-induced increases in granulocyte adhesion ability [45].

Administration of NPY in vivo reduced immune cell infiltration (especially neutrophils), regulated neutrophil phagocytosis, and induced nitric oxide (NO) and hydrogen peroxide (H_2_O_2_) production. In vitro, NPY and phorbol myristate acetate (PMA) were added simultaneously to the cell culture medium, in which NPY dose-dependently inhibited the oxidative burst of PMA-stimulated granulocytes. In contrast, NPY significantly reduced the production of granulocyte peroxidation products through Y2R/Y5R [48].

Dimitrijević et al. confirmed that the Y1R, Y2R, and Y5R subtypes in rat granulocytes change with age [49]. Their results showed that the percentage of Y 1R expressed by granulocytes in adult rats was significantly higher than that in younger and older rats, whereas there was no difference between the younger and older rats [49]. NPYRs have been found to exist on human neutrophils, and Y1R and Y2R are involved in phagocytosis [50]. The phagocytic function of fluorescently labelled *E. coli* was analysed using flow cytometry, showing a dose-dependent bimodal effect of NPY on neutrophil phagocytosis: low concentrations significantly enhanced phagocytosis and increased doses inhibited phagocytosis [50].

In vitro, NPY administered to human granulocytes activates Y1R and Y2R, which reduces their ability to phagocytise debris [50]. The antagonism of the Y1R antagonist BIBO3304 in vitro further underlines the role of the Y1R in the enhanced neutrophil adhesion induced by NPY. Interestingly, however, Y2R (BIIE0246) and Y5R (L152804) antagonists also blocked the potentiation of NPY, which may be related to their ability to inhibit granulocyte adhesion [45].

Furthermore, NPY induces neutrophil and macrophage chemotaxis [51, 52], attracting phagocytes to a site of infection. It can also regulate essential neutrophil functions, including phagocytosis of foreign pathogens and reactive oxygen species (ROS) production [50]. These results indicate that NPY influences granulocyte adhesion, phagocytosis, and inflammatory mediator release. NPY exerts its functions by binding to different receptors, which influences microenvironment activity and produces favourable effects on the body, thereby maintaining the microenvironment homeostasis.

#### Microglia

Microglia mainly exist in the CNS and regulate inflammation in the hypothalamus [53–59]. When stimulated by a pathogen, microglia release NO, ROS, inflammatory factors, and other cytotoxic substances, which induces inflammation.

Studies have indicated that NPY has neuroprotective effects both in vivo and in vitro [60–62]. Pain et al. first studied the neuroprotective effect of NPY in microglia-induced inflammation [63]. Microglial neuroinflammatory responses are regulated by Y1R activation, which induces an autocrine-like response [64]. Therefore, NPY is a potent inhibitor of microglial activation, which can be assessed by a change in microglial morphology from an activation-like state to a reactivation-like state [65]. Furthermore, NPY activation of Y1R effectively inhibited LPS and IL-1β-induced microglial motility and the opsonisation and phagocytosis of latex beads, mediated by downstream p38 signalling pathway activation [66]. Exogenous NPY administration reduced TNF-α-associated inflammation in Toll-like receptor (TLR2)-activated microglia [67], which was similar to an effect previously demonstrated on LPS-induced microglial growth factor-1β [65].

### NPY and cytokines

Cytokines are a general term for small immunomodulating proteins secreted by immune cells, and many have been shown to interact and synergise with NPY. Leukaemia inhibitory factor (LIF) can downregulate NPY expression in sympathetic neurons [68]. Interestingly, Wirth et al. discovered that in CNS neurons, LIF stimulated NPY expression [69]. Intracerebroventricular injections of IL-1β and TNF have been shown to promote anorexia and significantly reduce NPY mRNA expression [70, 71]. In the intestinal neurons of NPY knockout mice, TNF secretion was lower than in wild-type mice [72]. Also, dendritic cytokine factor 1 (DCF1) regulates NPY expression and maintains energy homeostasis. NPY expression was significantly reduced in the hypothalamus of DCF1 knockout (DCF1^−/−^, KO) mice [73].

During co-stimulation with toll-like receptor (TLR) agonists, NPY increased cytokine production, indicating that NPY enhances TLR-induced cytokine secretion [74]. Rosmaninho-Salgado et al. [75] suggested that IL1B is involved in the induction of NPY release. Park et al. demonstrated that NPY affected granulocyte colony-stimulating factor transduction, whereas mice lacking NPY showed diminished mobilisation of granulocyte colony-stimulating factor [76]. In brief, NPY can regulate the synthesis and release of various cytokines, which affect bodily behaviours.

## NPY and the nutritional microenvironment

Whether healthy or malignant, proliferating cells require a microenvironment that provides favourable conditions for their proliferation, differentiation, metabolism, and function. The microenvironment is influenced by local oxygen and other molecular gradients, tissue vascularisation, and cell metabolism [27], affecting local nutrient availability; an insufficiency of microenvironmental trophic and nutritional factors can retard cell growth and development. NPY can regulate nerve and blood vessel growth, promoting angiogenesis and neuronal structural integrity.

### NPY protects neurons from injury and promotes neurogenesis

NPY promotes the resolution of neuroinflammation, inhibits the release of proinflammatory cytokines, and attenuates the toxic effects of activated microglia [66, 77]. NPY and Y1R levels increase upon endotoxin-mediated activation of microglia [65]. This upregulation of NPY may be a feedback mechanism to counteract proinflammatory processes because activating Y1R can inhibit IL-1β and NO release [65]. Also, NPY reduces neuronal excitotoxicity and regulates calcium homeostasis. NPY selectively inhibits glutamatergic neurotransmission, which affects intracellular calcium flux and reduces oxidative stress and apoptosome formation [25]. Autophagy is an important process for tissue homeostasis, and a loss of autophagy leads to neurodegeneration, even in the absence of any disease-associated mutant protein [78]. NPY can stimulate autophagy in mouse and rat hypothalamic neural cells by activating Y1R and Y5R [79]. Additionally, an A1-40 mouse model treated with exogenous NPY demonstrated reduced oxidative stress, and mitochondrial dysfunction, via blockade of NO production and reduced lipid peroxidation [80, 81].

**Fig. 2:**
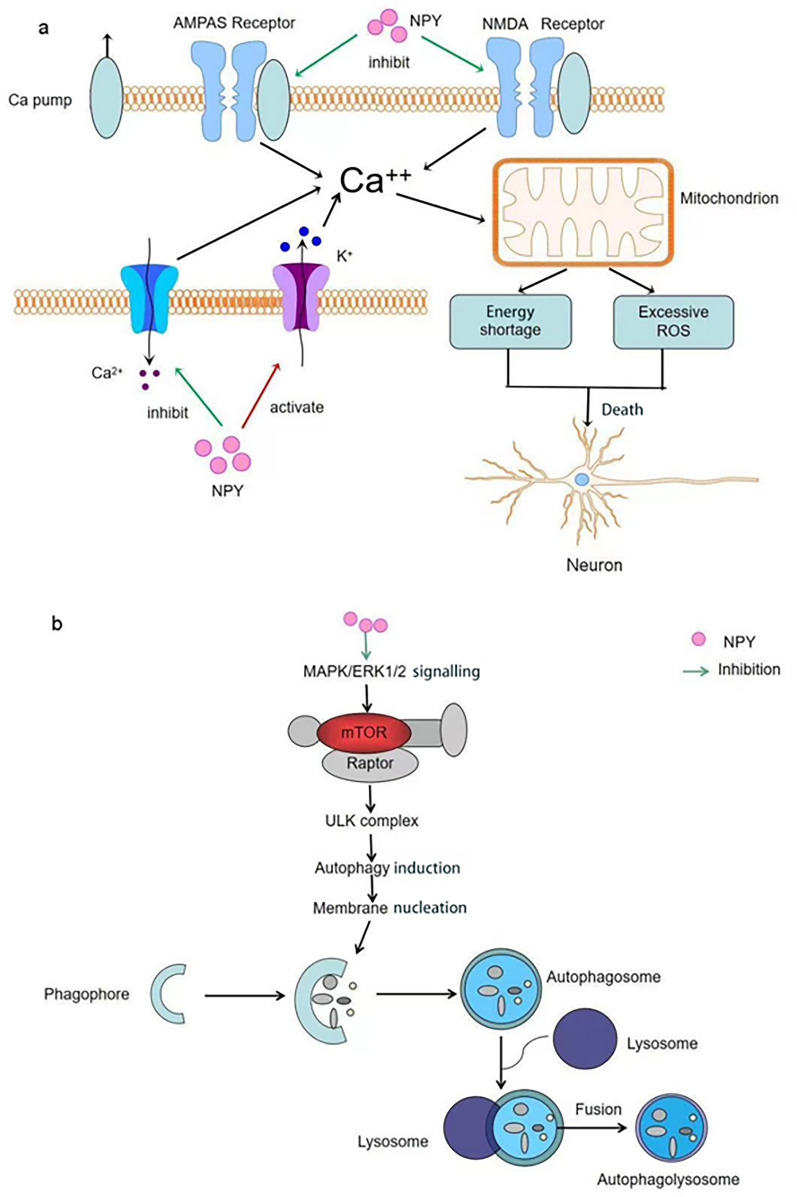
Neuroprotective effects of NPY. **a** NPY reduces calcium excess via activating potassium channels, inhibiting calcium channels, and inhibiting glutamate receptors, thereby avoiding mitochondrial dysfunction due to calcium excess, generating excessive ROS and ultimately causing neuronal death. **b** NPY induces autophagosome formation through the MAPK/ERK1/2 signalling pathway and stimulates lysosomes and phagocytic vesicles in cells to fuse into autolysosomes and remove harmful substances from the body

The neuroprotective effects of NPY include interaction with brain-derived trophic factors; for example, in Alzheimer's disease (AD), Aβ_25–35_ reduces intracellular nerve growth factor (NGF) and brain-derived neurotrophic factor (BDNF), whereas NPY fully restores the levels of these growth factors [82]. Taken together, NPY exerts neuroprotective effects through attenuating neuroinflammation, reducing excitotoxicity, inhibiting endoplasmic reticulum stress, oxidative stress, stimulating autophagy, and increasing nutritional support (Fig. 2).

### NPY interacts with BDNF to provide nutritional support

NPY regulates the expression of neurotrophic factors, which in turn promote the production of nutritional support. Neurotrophic factors (NTs) are a class of protein molecules produced by innervated tissues and Sertoli cells. NTs include NGF, BDNF, neurotrophin-3 (NT-3), and neurotrophin-4/5 (NT-4/5) [83]. They are essential for the growth and survival of neurons, for which they provide nutritional support.

In recent years, there have been studies on BDNF and its interaction with NPY. In vitro and in vivo studies have demonstrated that BDNF overexpression can significantly increase NPY mRNA and protein levels [84, 85]. Indeed, a small number of BDNF-overexpressing neurons rapidly cause upregulation of Trk receptor-dependent NPY mRNA in interneurons in thalamocortical co-cultured cortical explants [84]. Additionally, increasing BDNF levels can improve the motor phenotype and prevent cell atrophy and degeneration in HD transgenic mice [86].

Conversely, NPY overexpression also leads to the upregulation of BDNF mRNA levels [77, 87], and conclusions can be drawn from the experiments of Duarte-Neves et al. [77], who constructed an NPY overexpression model and injected adeno-associated viral (AAV) vectors encoding NPY or enhanced green fluorescent protein (EGFP) into transgenic mice to assess BDNF mRNA levels in a rodent model of Machado—Joseph disease striatum. It was found that NPY overexpression mediated 6.5-fold higher levels of BDNF mRNA in the mutant ataxin-3-transduced striatum at 4 weeks post-injection, compared with control EGFP co-transduction, indicating that overexpression of NPY promotes the generation of trophic support in striatal neurons [77]. In conclusion, NPY and BDNF cooperatively regulate the nutritional microenvironment and support neuronal development and survival. Neurotrophins act as a bridge, and NPY indirectly affects the trophic microenvironment through action on BDNF, thus providing trophic effects that support normal neuronal activity.

### NPY promotes angiogenesis and improves the microenvironment

**Fig. 3:**
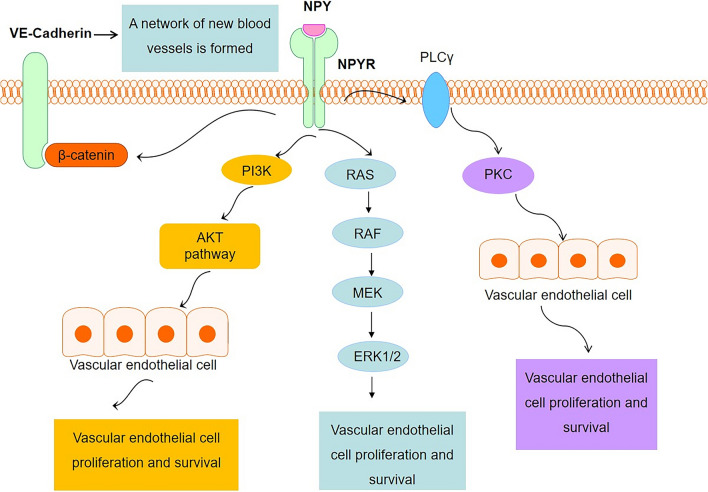
Effect of NPY on vascular endothelial cells. NPY promotes endothelial cell proliferation through the PI3K/AKT pathway, RAS/RAF/MEK/ERK1/2 pathway, and PKC pathway after binding to its receptor, thus providing nutrition for blood vessels and regulating the microenvironment

The effects of NPY on blood vessels include proliferation, development [88], and migration of neurons, smooth muscle, and endothelial cells (Fig. 3). In 1998, Zukowska et al. discovered that NPY is a potent pro-angiogenic factor, and its effect is equivalent to that of fibroblast growth factor (bFGF) and vascular endothelial growth factor (VEGF) [89]. Studies have found that NPY at physiological concentrations can stimulate endothelial cell proliferation, migration, and capillary angiogenesis by activating Y1R and Y2R on endothelial cells, promoting vascular endothelial sprouting, migration, adhesion, and the formation of new blood vessels [90]. It was also demonstrated that Y1R, Y2R, and Y5R antagonists affect endothelial cell proliferation (Y1R + Y2R, Y1R + Y5R, Y2R + Y5R) and capillary formation of stromal cells (Y1R + Y2R + Y5R) [91].

Furthermore, NPY promotes the secretion of NO and VEGF from vascular endothelial cells, reduces the production of endothelin-1 [92] and angiostatin, and promotes the proliferation and migration of endothelial cells, all of which promote angiogenesis [93]. VEGF is the main chemokine that induces angiogenesis [94]. Like endothelial cells, VSMCs are core components of blood vessels. NPY promotes the migration and proliferation of VSMCs in female rats, whereas NPY administered at 10^−6^ mol/L effectively regulated cell migration and proliferation, with upregulation of both Y1R and Y5R [95]. NPY has been shown to promote VSMC growth through the interaction of various signalling pathways such as calcium/calmodulin-dependent kinase II (CaMKII), protein kinase C (PKC), and mitogen-activated protein kinase (MEK1/2) [90]. In conclusion, NPY improves the nutritional microenvironment of the body by promoting the formation of blood vessels.

## Applications of NPY in diseases

**Fig. 4:**
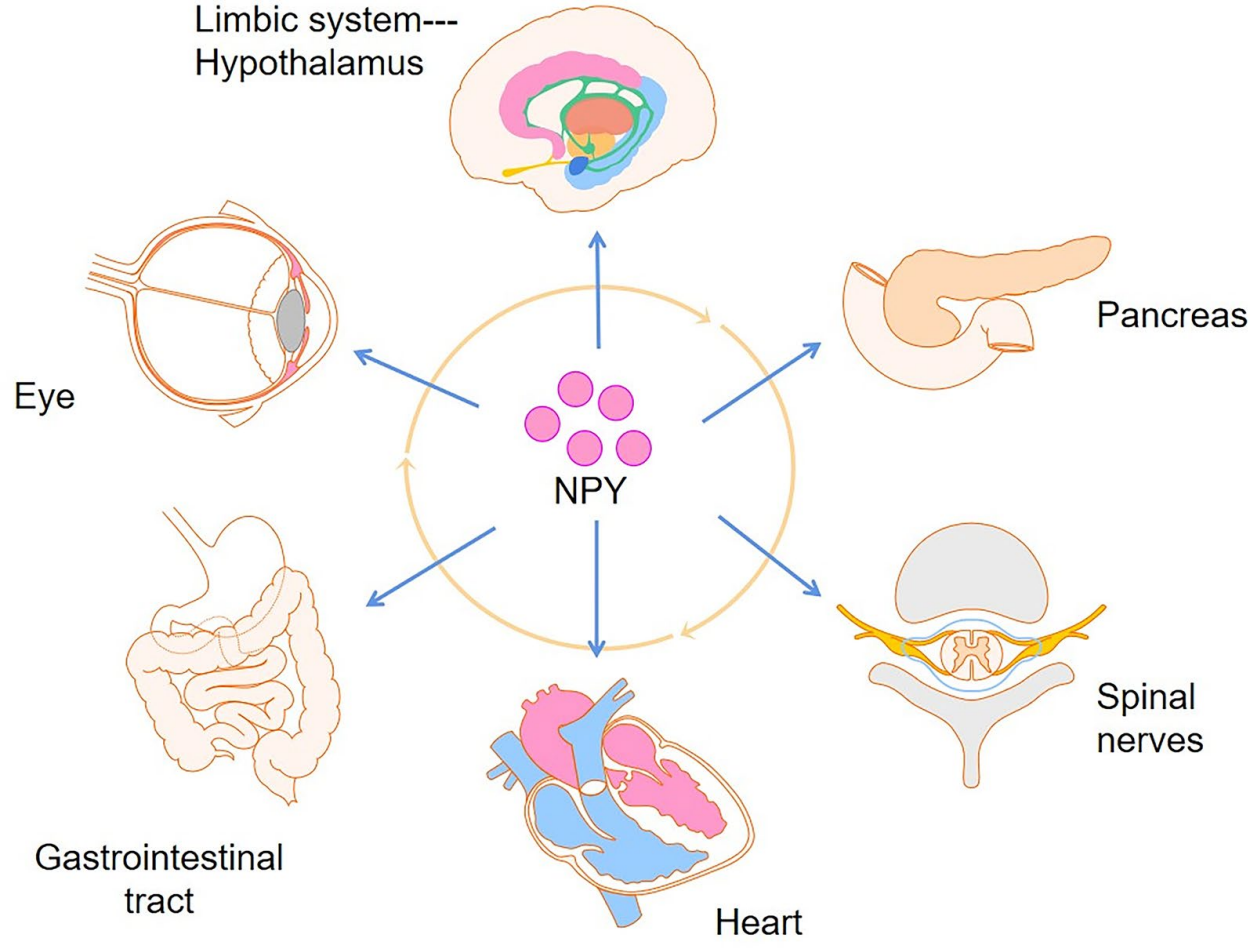
Human diseases associated with NPY. NPY is a pleiotropic molecule that influences cell proliferation, cardiovascular and metabolic function, pain, and neuronal excitability. NPY is widely distributed and functions in various parts of the body. Therefore, if NPY is out of balance, it causes systemic diseases, including those of the eyes, and of the nervous, digestive, and cardiovascular systems

NPY is associated with various diseases, including CNS diseases, cardiovascular diseases, obesity, adipose tissue inflammation, autoimmunity, and atherosclerosis. The pathogenesis of these diseases depends on the tissue microenvironment and the stimuli it receives, which may be influenced by NPY (Fig. 4).

### Endocrine and metabolic diseases including obesity

NPY is an important regulator of feeding behaviour [96]. When NPY production in the hypothalamus and dorsal medial nucleus is abnormally high, it can promote obesogenic food-seeking behaviour. Ip et al. constructed a central amygdala (CEA)-specific NPY overexpression model; male Npy^Cre/+^ mice were injected with the AAV-flex-NPY vector for the CEA, and changes in body weight, food intake, and energy expenditure (EE) were recorded [97]. The level of NPY mRNA in the amygdala of mice injected with the AAV-flex-NPY vector was significantly higher than that of control mice. Importantly, the overproduction of NPY in the medial nuclei of the CEA resulted in weight gain due to increased calorie intake, indicating that increased food intake due to overexpression of NPY has a specific effect on fat metabolism. NPY in CEA neurons is a key neurotransmitter mediating increased food intake. In addition, the team injected an AAV-flex-^Leu31, Pro34^NPY viral construct (flex-L/P/-NPY), which prefers Y1R, into mice, and observed a significant increase in body weight. Interestingly, preferential activation of the Y1R signalling pathway resulted in significantly lower EE compared with that in endogenous NPY overexpression. Therefore, the use of ^Leu31, Pro34^NPY variants suggests that the Y1R response pathway is activated by CEA-derived NPY neurons under stress [97]. Other studies have reached the same conclusion: NPY mRNA and protein expression in the hypothalamus of obese animals is increased, compared with that of animals with normal weight [98]. NPY promotes obesity after binding Y1R and Y2R expressed within white adipose tissue (WAT), which promotes WAT proliferation [99]. A study showed that NPY promoted blood vessel formation in abdominal WAT by upregulating Y2R expression on the anterior surface of WAT, increasing the proliferation and differentiation of WAT cells, leading to pathogenic adipose accumulation and obesity [100–102].

Additionally, both central and peripheral sources of NPY can induce obesity by promoting the accumulation of WAT throughout the body [103, 104]. Therefore, NPY is activated in adipose tissue, increasing lipogenesis and angiogenesis, leading to abdominal obesity and metabolic syndrome. In addition to NPY itself, the mechanisms causing obesity also include the inflammatory response, in which macrophages play a role. Studies have shown that macrophage accumulation in adipose tissue is positively correlated with obesity-related inflammation [105]. Increased recruitment of macrophages to adipose tissue in obese patients leads to the secretion of various proinflammatory cytokines [106, 107]. These proinflammatory cytokines may contribute to the differentiation of adipose precursor cells, further exacerbating the increased adipose accumulation in obesity [108].

In summary, NPY and the activation of its receptors can promote macrophage infiltration of adipose tissue and the proliferation and differentiation of adipocytes, ultimately contributing to the development of obesity and metabolic syndrome, in which NPY regulates the inflammatory and nutritional microenvironment.

### Neurodegenerative diseases (Alzheimer's disease and Huntington's disease)

AD is the most common type of senile dementia, and its pathogenesis may be induced by a variety of microenvironmental factors [109], such as abnormal deposition of β-amyloid (Aβ) protein between neurons and the formation of neurofibrillary tangles caused by excessive phosphorylation of tau protein in neurons. Together, these result in chronic neuroinflammation and mitochondrial dysfunction. Microenvironmental disorders caused by amyloid plaques and neurofibrillary tangles are the most significant causes of AD pathogenesis [110, 111].

Studies have shown that in AD transgenic mice models, NPY mRNA expression is decreased in the hippocampus and cerebral cortex [112]. In vitro, amidated NPY protects cultured neural cells from the neurotoxic effects of Aβ [113], which may protect against neurotoxicity by activating Y2R in the hippocampus rather than simply representing the end products of hydrolysis. In addition to counteracting the toxic effects of Aβ, NPY can also restore neurotrophin levels in neuroblastoma cells. An SH-SY5Y neuroblastoma cell line was exposed to toxic concentrations of Aβ_25-35_ peptide fragment (Aβ_25-35_), and neurotrophin expression was measured before and after NPY preincubation. The toxic effects of Aβ_25–35_ were completely abolished after 24 h, and both NGF and BDNF protein levels were significantly higher in cells pretreated with NPY than in cells exposed to Aβ_25–35_ alone [114]. Moreover, Y1R and Y2R are both expressed in the hippocampal microglia. Hippocampal microglia are the most important immune cells in the central nervous system and play an important regulatory role in the pathogenesis of AD. In methamphetamine-induced microglia death, Y2R activation promotes the survival of microglia [115]. However, Y1R activation inhibits microglia activation [65]. The effects of Y1R and Y2R receptors on microglia appear to be contradictory, indicating an evolutionarily conserved mechanism of autoregulation. The above reports illustrate that NPY may be used as a neuroprotective agent and a drug to treat AD.

Parkinson’s disease and Huntington’s disease are devastating CNS diseases, involving degeneration of dopaminergic neurons in the substantia nigra and striatum. As in Alzheimer’s disease, NPY plays a vital role in both these diseases. In 1986, Kerkerian et al. observed that in an animal model of Parkinson’s disease, the absence of dopaminergic neurons in the substantia nigra striatum resulted in a significant increase in the number of NPY-expressing cells in the striatum [116].

In vivo, the neuroprotective effects of NPY on the dopaminergic neurons in the substantia nigra and striatum was blocked by the Y2R antagonist BIIE0246, suggesting that the effects of NPY are mediated through Y2R and may involve the activation of mitogen-activated protein kinase and Akt pathways [117]. Also, NPY and the Y2R agonist NPY (3–36) have been shown to protect PC12 cells expressing mutant huntingtin (HTT) exon-1 against mutant HTT-induced cell death [118], suggesting that NPY exerts multiple protective effects on the CNS. These protective effects mainly involve regulating immune cells, cytokine release, enzymes, and proteins in the brain microenvironment to protect nerve cells from damage, restore glial cell function, and provide sufficient nutrition for neurons.

### Cardiovascular disease (myocardial infarction)

NPY is released by sympathetic nerves, which can activate Y1R, causing vasoconstriction, which can synergise with norepinephrine. Therefore, NPY plays a crucial role in the cardiovascular system. NPY is associated with atherosclerosis, coronary heart disease [119–121], and pulmonary hypertension [122]. Many factors affect atherosclerosis, and ischemic diseases often occur after their onset. Endothelial dysfunction is a major feature that can lead to atheromatous plaque formation. NPY can promote the mitosis of endothelial and VSMCs, which contributes to intimal thickening [123, 124] and is essential for the formation of capillaries. The pro-angiogenic effect of NPY may be the main cause of plaque rupture and haemorrhage. In vitro studies of isolated cardiomyocytes have shown that NPY has variable and transient effects, including stimulating hypertrophy in cardiomyocytes [125], and can act as a powerful vasoconstrictor that enhances the vasoconstrictive effects of norepinephrine, causing increased blood pressure, local vascular stenosis, and heart spasm, which may eventually lead to myocardial ischemia and infarction. Clinical studies have confirmed that elevated plasma NPY levels are associated with myocardial ischemia and infarction [121, 126], and NPY levels can be used to inform disease severity and prognosis.

Studies have shown that intracoronary injection of NPY in patients with angina pectoris may cause myocardial ischemia, which manifests as chest pain and an abnormal electrocardiogram result [127]. Plasma NPY levels are increased in patients with acute myocardial infarction or angina, associated with tachycardia and left ventricular failure [126]. Also, in the chronic hypoxic mouse model, Y1R was upregulated, whereas expression of both NPY and Y1R was increased in the lungs of monocrotaline and SU5416-hypoxia rats, on a functional level [122]. Collectively, the effect of NPY on cardiovascular diseases is mainly through the regulation of neurons in neurohumoral microenvironments.

Meanwhile, NPY acts in conjunction with norepinephrine, causing vasoconstriction, increased peripheral resistance, vascular stenosis, and disruption of the vascular microenvironment’s homeostasis. Conversely, it acts as a local transmitter and trophic factor to affect blood vessel function. NPY can act as a neurotransmitter with effects similar to those of VEGF, promoting the proliferation, growth, and migration of endothelial cells and VSMCs.

### Stem cell therapy

Stem cells are pluripotent cells with tissue-restorative properties that replace dead cells, help restore damaged tissue, secrete a variety of bioactive factors, promote angiogenesis, and regulate immune responses [128, 129]. Stem cell therapy has been successfully applied in the clinical treatment of various diseases, including diabetes [130], spinal cord injury, and osteoporosis [131]. Here, we discuss the effects of NPY regulating vital activities, such as proliferation, differentiation, and migration of stem cells, and the mechanisms involved. This, in turn, provides more theoretical support for stem cell therapy in clinical diseases.

At present, the vast majority of studies has demonstrated that NPY plays a role within the bone through Y1R [132], promoting the proliferation, migration, and differentiation of bone marrow stem cells (BMSCs), and preventing osteoblast apoptosis [133, 134], and thereby promoting fracture healing. However, a few results of in vitro cell culture experiments have shown that NPY inhibits BMSC osteogenesis, increases the RANKL/OPG ratio, and downregulates the expression of cAMP, p-PKAs, and p-CREB, whereas the Y1R antagonists inhibit the effect of NPY [135]. Therefore, Y1R may inhibit the differentiation of BMSCs into osteoblasts through the cAMP/PKA/CREB pathway.

In addition to their effects on the bone marrow, it has been reported that NPYRs are highly expressed in hematopoietic stem cells (HSCs). NPY is necessary for HSC survival and bone marrow homeostasis, and it plays a key role in the proliferation and mobilisation of HSCs [136, 137]. Park et al. discovered that HSC mobilisation was impaired in NPY-deficient mice, and that this was ameliorated by endogenous NPY [76]. This mobilisation was achieved by reducing HSC maintenance factors and regulating matrix metalloproteinase-9 (MMP-9) activity in osteoblasts through Y1R [76]. Ulum et al. revealed the direct effect of NPY on HSCs [137]. They found that NPYRs were present on both immature and mature hematopoietic cell subsets, NPY tended to inhibit HSC proliferation when used at relatively high concentrations and had a consistent, although not significant, effect on HSC arrest at the G0 phase of the cell cycle [137]. They also used RT-qPCR to detect the expression levels of the FOXO3, DICER1, SMARCA2, and PDK1 genes (involved in quiescent stem cell characteristics) in NPY-treated control HSCs. Their results showed that these four specific genes were significantly upregulated. The above data indicate that NPY may be directly involved in regulating the quiescence of HSCs, in addition to its role in the maintenance and migration of HSCs [137].

Alternatively, there are few studies on NPY and adipose-derived stem cells (ADSCs), but it has still been shown that Y2R and Y5R are expressed in human ADSCs, and NPY may play a role in human ADSC proliferation and adipogenic differentiation by interacting with these two receptors [138]. Liu et al. established a model of human ADSCs from human adipose tissue and differentiated them into adipocytes at different concentrations of NPY to determine the effects of different NPY doses on proliferation and adipogenic differentiation [138]. In their study, low-dose NPY treatment promoted the proliferation of human ADSCs, while high-dose treatment inhibited it. NPY significantly promoted lipid accumulation, increased the size of lipid droplets, and increased the levels of adipocyte markers PPAR-γ and C/EBPα [138]. In conclusion, the prospective applications of NPY treatment of stem cells are promising. However, there are unexplored mechanisms induced by NPY that need to be resolved before these treatments can be utilised in the clinic.

## Conclusions

NPY is produced by various cells and is widely expressed throughout the body, stimulates immune responses, promotes angiogenesis, and regulates tissue microenvironments. There is a synergistic effect between different subtypes of NPYRs. At present, there are many reports on Y1R and Y2R, but further studies on Y4R and Y5R are required in the future. NPY has potential applications in treating diseases. In particular, NPY has auxiliary effects in stem cell applications. NPY can be used as an adjunct to stem cell transplantation and can promote tissue regeneration when appropriate, which is a positive implementation of stem cell treatment of diseases. However, this research is still in its infancy, and the specific mechanisms of different diseases are not well understood; further research is necessary.

## Data Availability

Not applicable.

## Abbreviations

AD: Alzheimer’s disease
ADSCs: Adipose-derived stem cells
BDNF: Brain-derived trophic factor
BMSCs: Bone marrow stem cells
CEA: Central amygdala
DCF1: Dendritic cytokine factor 1
EE: Energy expenditure
EGFP: Enhanced green fluorescent protein
HSCs: Hematopoietic stem cells
IL: Interleukin
LIF: Leukaemia inhibitory factor
LPS: Lipopolysaccharide
NGF: Nerve growth factor
NPY: Neuropeptide Y
NPYRs: NPY receptors
NT-3: Neurotrophin-3
NTs: Neurotrophic factors
PTX3: Pentraxin 3
TLR: Toll-like receptor
TNF: Tumour necrosis factor
VEGF: Vascular endothelial growth factor
VSMCs: Vascular smooth muscle cells
TGF: Transforming growth factor
